# Effects of supplementing late-gestation sow diets with zinc on preweaning mortality of pigs under commercial rearing conditions^1^

**DOI:** 10.1093/tas/txaa010

**Published:** 2020-01-29

**Authors:** Julia P Holen, Pedro E Urriola, Mark Schwartz, Jae-Cheol Jang, Gerald C Shurson, Lee J Johnston

**Affiliations:** 1 Department of Animal Science, University of Minnesota, St. Paul, MN; 2 Schwartz Farms Inc., Sleepy Eye, MN; 3 West Central Research and Outreach Center, University of Minnesota, Morris, MN

**Keywords:** carcass characteristics, mortality, preweaning, swine, zinc

## Abstract

The objective of this experiment was to determine preweaning survival of pigs when sows were supplemented with 3 dietary levels of zinc (Zn) in late gestation. Gilts and sows (*n* = 339) were assigned to 1 of 3 dietary treatments based on parity. Dietary treatments were 1) Control—sows fed a corn–soybean meal-based diet containing 125 ppm total supplemental Zn supplied by ZnSO_4_ (75 ppm Zn) and AvailaZn (50 ppm Zn, CON); 2) Intermediate—as Control + 240 ppm supplemental Zn as ZnSO_4_ (INT); and 3) High—as Control + 470 ppm supplemental Zn as ZnSO_4_ (HI). Final supplemental Zn concentrations of the 3 dietary treatments were 1) CON—125 ppm; 2) INT—365 ppm; and 3) HI—595 ppm. Sows received dietary treatments from about day 85 of gestation until farrowing. Individual piglet birth weights were recorded within 12 h of parturition. Instances of piglet mortality were recorded daily. The statistical model considered fixed effects of treatment and random effects of parity. Piglets from sows fed the INT diet had heavier (*P* < 0.05) birth weights than those fed CON (1.42 vs. 1.38 kg, respectively), while offspring from sows fed HI tended to have heavier (*P* < 0.10) birth weights (1.40 kg) than pigs from INT sows. Furthermore, incidence of low birth weight pigs was less (*P* < 0.05) for sows consuming INT compared with sows fed CON and HI. Despite differences in birth weight, there were no differences (*P* > 0.05) in total pigs born, born alive, or weaned, nor differences in individual piglet gain or weaning weight across treatments. Mortality of low birth weight pigs was lowest (*P* < 0.05) for offspring from sows fed HI (28.1%) compared with offspring from sows fed INT (36.1%) and CON (38.3%). Similarly, overall piglet mortality tended to decrease (*P* < 0.10) as dietary Zn content increased (CON: 15.0%, INT: 13.2%, and HI: 12.2%). A subset of pigs (*n* = 420, *n* = 140/treatment) were selected at weaning to evaluate effects of dietary treatment on postweaning performance. There were no significant effects of sow Zn supplementation on final body weight, days to market, or carcass characteristics of market pigs. Overall, effects of supplemental dietary Zn at 365 and 595 ppm in late gestation improved preweaning survival of low birth weight piglets and reduced overall preweaning mortality of piglets.

## INTRODUCTION

Sows in transition from gestation to lactation are in a period of dramatic physiological change due to high nutrient demands of rapidly growing fetuses *in utero*, parturition, and lactation. These physiological changes require coordinated hormonal, nutritional, and management transitions to optimize piglet viability and postnatal growth (Theil, 2015). Inadequate preparation for these massive physiological changes could lead to increased stillbirth rate, increased number of low birth weight pigs (<1 kg birth weight), and increased preweaning mortality of piglets. Preweaning mortality can be attributed to many factors such as low viability, trauma from crushing, starvation, or disease (Vaillancourt et al., 1990; Lay et al., 2002; Edwards and Baxter, 2015). Commercial swine farms commonly experience preweaning mortality rates between 12% to 25% (Alonso-Spilsbury et al., 2007; Nuntapaitoon and Tummaruk, 2018; PigCHAMP, 2019).

Birth weight significantly influences lifetime growth performance and subsequent carcass characteristics of market pigs (Lay et al., 2002; Douglas et al., 2013). Piglets weighing less than 1 kg at birth typically are at greater risk for mortality and poor lifetime growth performance compared with pigs with normal birth weights (Foxcroft et al., 2006; Calderon Diaz et al., 2017). Therefore, sows that produce pigs with birth weights greater than 1 kg have greater economic value for pork producers because they have enhanced postnatal piglet survival and pig growth performance. Postnatal management, environment, and nutritional interventions to improve piglet viability and growth have resulted in varying degrees of success (Deen and Bilkei, 2004; Douglas et al., 2014; Edwards and Baxter, 2015). A more effective strategy might be to intervene prenatally to better prepare piglets for life outside the sow’s uterus.

Increasing dietary zinc supplementation may be a useful prenatal intervention. Previously, zinc deficiency has been associated with intrauterine growth retardation, subsequent decrease in birth weight, lack of competent immune and neurological system development, and increased preweaning mortality in rats and humans (Hurley et al., 1973; Simmer and Thomson 1985). In swine, elevated dietary zinc concentrations can reduce the incidence of stillborn pigs (Hill et al., 1983) and increase litter birth weight (Payne et al., 2006). Researchers have demonstrated that zinc, copper, and manganese accumulate in high concentrations in the conceptus (Hostetler et al., 2003). Furthermore, after day 90 of gestation, maternal liver zinc concentrations decrease, yet, fetal liver zinc increases (Hostetler and Kincaid, 2004). Impaired accumulation of these trace minerals may negatively affect the piglet’s chance of survival. Vallet et al. (2014) demonstrated, with a limited number of gilts (*n*=56), that elevated dietary zinc concentration during late gestation reduced preweaning mortality of low birth weight pigs, but this observation has not been verified under large-scale commercial conditions. Therefore, we hypothesized that preweaning survival and lifetime performance of pigs weighing less than 1 kg at birth would improve by feeding sows increasing levels of dietary Zn during the last 30 d prepartum. Our objective was to evaluate this hypothesis under commercial conditions present in a large-scale sow farm.

## MATERIALS AND METHODS

The experimental protocol was reviewed and approved by the University of Minnesota Institutional Animal Care and Use Committee (IACUC# 1083-35724A). The experiment began in May 2018 and concluded in February 2019. This experiment was conducted in a commercial sow facility (1,200 sows; Schwartz Farms, Inc.) located in Comfrey, MN.

### Animals, Housing, and Treatments

Three consecutive weeks of production that included 339 females (parity 0 to 7; PIC Camborough, Hendersonville, TN) were assigned based on parity to 1 of 3 dietary treatments at 85 ± 3 d of gestation.

Dietary treatments consisted of 1) Control—sows fed a corn–soybean meal-based diet containing 125 ppm total supplemental Zn supplied by ZnSO_4_ (75 ppm Zn) and amino acid complexed Zn (50 ppm Zn; AvailaZn; Zinpro Corp., Eden Prairie, MN; CON); 2) Intermediate—as Control + 240 ppm supplemental Zn as ZnSO_4_ (INT); and 3) High—as Control + 470 ppm supplemental Zn as ZnSO_4_ (HI). Final supplemental Zn concentrations of the 3 dietary treatments were 1) CON—125 ppm; 2) INT—365 ppm; and 3) HI—595 ppm. Composition of gestation and lactation diets was based on the farm’s standard operating procedures (Table 1). Dietary treatments were imposed by adding 60 mL (45 g; INT) or 120 mL (90 g; HI) of the Zn supplement to feed hoppers once daily for sows assigned to INT or HI treatments, respectively (Table 2). The Zn top-dress provided 519 mg or 1,038 mg of additional Zn per day, respectively, for sows assigned to INT and HI treatments. Control sows did not receive any top-dressed Zn supplement. Sows remained on their assigned dietary treatment and received 2.2 kg of feed once daily until parturition. Immediately after parturition, all sows were fed a common lactation diet and allowed *ad libitum* access to feed and water.

**Table 1.** Ingredient and nutrient composition of sow diets (as-fed basis)

Ingredient, %	Gestation	Lactation
Corn	49.20	52.90
Wheat middlings	15.00	—
Soybean meal	2.50	26.96
DDGS^1^	30.00	15.00
Choice white grease	—	1.00
Limestone	1.70	1.50
Monocalcium phosphate, 21% P	0.45	0.80
Salt	0.45	0.45
l-Lysine HCl	0.23	0.32
l-Threonine	—	0.10
Choline chloride 60%	0.14	0.05
Dyna K	—	0.63
Sow pack^2^	0.08	0.04
Premix^3^	0.25	0.25
Total	100.00	100.00
Analyzed nutrient composition		
Moisture, %	13.4	15.7
Crude protein, %	16.0	19.2
Crude fat, %	4.3	4.3
Crude fiber, %	3.6	2.1
Ash, %	6.0	8.0
Calcium, %	1.01	1.94
Phosphorus, %	0.63	0.60
Zinc total, ppm	184.6	255.9

^1^Dried distillers grains with solubles.

^2^Contained direct-fed microbial, mycotoxin binder, yeast culture, and carnitine.

^3^Contained the following nutrients per kg of premix: vitamin A, 4,409,240 IU; vitamin D_3_, 1,587,326 IU; vitamin E, 26,455 IU; menadione, 1,764 mg; riboflavin, 3,307 mg; niacin, 19,842 mg; pantothenic acid, 13,228 mg; pyridoxine, 5,732 mg; vitamin B_12_, 15 mg; folic acid, 661 mg; biotin, 88 mg; phytase, 132,277 FTU; zinc, 110,231 ppm (60% as ZnSO_4_, 40% as AvailaZn, Zinpro, Eden Prairie, MN); iron, 97,003 ppm; manganese, 35,274 mg; chromium, 176 ppm; copper, 14,550 ppm; iodine, 485 ppm; selenium, 265 ppm.

**Table 2.** Ingredient and zinc composition of top-dress (as-fed basis)

Ingredient, %	Top-dress
Corn	95.8
Choice white grease	1.0
Zinc sulfate, monohydrate	3.2
Total	100.0
Analyzed composition	
Zinc total, ppm	11,530

Sows were housed individually in stalls during gestation on partially slatted concrete floors. Feed for sows was dropped once daily into a trough at the front of each stall that was connected to adjacent stalls. Because of this connected trough, treatments were assigned to a block of gestation stalls to avoid cross-contamination of treatments from 1 sow to adjacent sows. One “buffer” sow was placed at the end of each block of stalls to receive the same dietary treatment but was not included in the experiment. At about day 110 of gestation, sows were moved to individual farrowing stalls within farrowing rooms until weaning of litters. Each farrowing room contained 39 stalls. Farrowing stalls were equipped with 1 stainless steel feeder and 1 nipple waterer on a partially slatted floor over a deep manure collection pit. An independent controller within each farrowing room operated all heaters and ventilation fans. One heat lamp was placed in the creep area of each farrowing stall as a supplemental heat source for piglets.

### Sow and Piglet Performance

Sows were identified individually using ear tags. Body condition and lameness scores were recorded at initiation of dietary treatments, at about day 110 of gestation, within 24 h of expected parturition, and at weaning. Body condition scores were determined using a body condition caliper placed at the last rib of the sow (Knauer and Baitinger, 2015). Visual lameness scores were recorded and assigned as sows stood up within stalls according to the following scale: 1) Normal: sow standing with weight equally distributed on all feet; or 2) Lame: sow with arched back, weight unequally distributed on feet, and difficulty or inability to stand.

Sow reproductive performance measurements included total number of piglets born, born alive, stillborn, mummified, and weaned per litter. Within 12 h of birth and prior to cross-fostering, all piglets were weighed individually and ear tagged. In some circumstances, although uncommon, ear tags were not retained by piglets. As a result, only pigs that retained ear tags throughout the entire lactation period were included in the final data analysis to ensure correct piglet identification. Ear tags were color-coded to match that of their dam’s assigned dietary treatment. Litter sizes were standardized to 12 or 13 piglets per sow by cross-fostering within 24 h of farrowing. Cross-fostering of piglets within assigned gestation dietary treatments was attempted, but not strictly controlled throughout lactation. All piglets were processed according to the standard operating procedure established by the farm within 24 to 48 h of birth. Piglet processing included tail docking, needle teeth clipping, administering injectable iron, and castration of male piglets. Incidence of stillborn and mummified piglets was recorded at birth but birth weight of these piglets was not recorded. Any pigs that died shortly before or during parturition, due to asphyxia or dystocia, were classified as stillborn. Piglets were monitored daily for instances of morbidity and mortality. Any dead piglets were weighed and date of death, piglet eartag identification, sex, and treatment were recorded. One day before weaning, individual piglet body weights were determined and recorded to calculate total weight gain during the nursing period. Piglets were weaned at about 18.1 ± 0.1 d of age.

A subset of about 15 litters per treatment (*n* = 140 pigs/treatment) of both low birth weight (*n* = 30 pigs/treatment) and normal to heavy birth weight (*n* = 110 pigs/treatment) pigs were selected at weaning to monitor postweaning growth performance and subsequent carcass characteristics. Selected pigs were fed common, corn–soybean meal-based diets that met or exceeded NRC (2012) requirements throughout the entire growing-finishing period. All instances of mortality were recorded. Pigs were tattooed individually before shipment and were harvested at JBS USA Holdings, Inc. (Worthington, MN). Hot carcass weight, backfat depth measured between the third and fourth rib, and loin depth were recorded for each pig. An optical probe (Fat-O-Meat’er, Frontmatec Group, Denmark) was used to determine backfat thickness and loin depth of all carcasses. The following equation, as determined by JBS USA, was used to calculate percentage carcass lean: Percentage carcass lean = 58.86 − 0.61 × (backfat depth, in.) + 0.12 × (loin depth, in.). The following equations reported by NPPC (2000) were used to calculate percentage fat-free carcass lean, and lean gain per day: Percentage fat-free carcass lean (FFL) = [15.31 − (31.277 × backfat depth, in.) + (3.813 × loin depth, in.) + (0.51 × hot carcass weight, pounds)]/hot carcass weight × 100; and Lean gain/day = (FFL at ending weight − FFL in feeder pig)/days on test.

### Sample Analysis

Two random samples of the zinc top-dress and gestation diet were collected at initiation and throughout feeding of dietary treatments to each farrowing group. One random sample of the lactation diet was collected at the midpoint of the trial. All samples were stored at −20 °C until shipment for analysis. Diet and top-dress samples were sent to Minnesota Valley Testing Laboratories, Inc. (New Ulm, MN) for proximate analysis and determination of zinc concentration. Standard procedures (AOAC International, 2006) were followed for analysis of moisture (Method 930.15), ash (Method 942.05), crude fat (Method 2003.05), crude fiber (Method BA6A-05), crude protein (Method 990.03), calcium (Method 985.01), phosphorus (Method 985.01), and zinc (Method 985.01) concentrations.

### Statistical Analysis

Experimental data were analyzed using the PROC GLIMMIX procedure of SAS (Version 9.4, SAS Institute Inc., Cary, NC) with a Gaussian distribution. Sow was considered the experimental unit. Postweaning data considered pig as the experimental unit. The statistical model included fixed effects of dietary treatment, farrowing group (cohorts that farrowed within 1 wk), and their interaction. Farrowing group was tested as a fixed effect for all variables but did not influence performance or mortality variables, so it was removed from the final statistical model. Treatment means were separated using the PDIFF option with the Tukey–Kramer adjustment for multiple comparisons.

Chi-square analyses were used to determine the influence of gestation dietary treatments on categorical response variables such as preweaning and postweaning piglet mortality, lameness scores, and incidence of stillbirths and mummies. Pigs were categorized into Low (< 1.00 kg), Normal (1.01 to 1.75 kg), and Heavy (> 1.76 kg) birth weight groups. The threshold of 1.0 kg was selected to define Low birth weight pigs because piglets weighing less than 1.0 kg at birth experience increased pre-wean mortality than heavier littermates at birth (Calderon Diaz et al., 2017; Zeng et al., 2019). Furthermore, 1.0 kg represented one standard deviation below the mean birth weight of all piglets observed in the study. The Heavy birth weight threshold was selected because it represented one standard deviation above the mean birth weight of piglets in the study. All data were reported as least square means and considered statistically significant at *P* < 0.05 with *P* < 0.10 considered a trend.

## RESULTS AND DISCUSSION

### Sow Performance

Average sow parity and days of feeding supplemental Zn were not different across dietary treatments (Table 3). Average gestation length was greater (*P* < 0.05) for HI sows compared with sows fed INT and CON. However, lactation length and days to first service were not different across treatments. At initiation of the experiment, body condition caliper scores were lower (*P* < 0.05) for CON sows compared with sows fed INT and HI diets. Body condition scores 1 d before farrowing and at weaning were not different across treatments. Sow farrowing performance including total number of pigs born, born alive, and weaned per litter was not different regardless of dietary treatment throughout the experiment. Sows farrowed about 14 total pigs per litter, of which 13 to 14 pigs were born alive, and sows averaged slightly less than 11 piglets weaned per litter. Sows utilized in this experiment performed similar regarding litter size farrowed and weaned to that of many US commercial swine production systems (Knox et al., 2013; Stalder, 2018). Instances of lameness throughout the experiment were rare and ranged from 0% to 2% among the 3 treatments at 3 assessment points (day 85 of gestation, 1 d before expected parturition, and weaning). Incidence of lameness was not different across treatments. The National Pork Board’s Pork Quality Assurance Plus program (PQA Plus, version 4.0; National Pork Board, 2019) set the acceptable incidence of severe lameness at 2%. Therefore, sows involved in this experiment displayed an incidence of lameness at or below the threshold set by the PQA Plus program. When further evaluating farrowing performance of sows, there were no differences across treatments regarding the total number of stillborn or mummified pigs (Table 4).

**Table 3.** Effect of supplemental Zn in late gestation on farrowing performance of sows

	Treatment				
Item	CON^1^	INT^2^	HI^3^	SE	*P*-value
No. of sows	112	112	115	—	—
No. of litters	108	104	110	—	—
No. of piglets	1,565	1,424	1,525	—	—
Parity	2.9	3.0	2.9	0.2	0.92
Days fed supplemental Zn	35.8	36.0	36.1	0.4	0.70
Gestation length, d	115.2^a^	115.2^a^	115.6^b^	0.1	<0.01
Lactation length, d	22.4	21.9	22.3	0.3	0.45
Days to service	6.9	7.1	5.8	0.9	0.43
Sows mated within 7 d postweaning^4^, %	85.9	83.3	89.8	—	0.48
Body condition score^5^					
Day 85 gestation^6,7^	14.9^a^	15.5^b^	15.5^b^	0.2	0.03
Prefarrow^8^	13.1	13.3	13.3	0.6	0.68
Weaning	11.4	11.8	11.8	0.4	0.32
Farrowing performance					
Total pigs born/litter	14.7	13.8	14.2	0.4	0.23
Pigs born alive/litter	14.0	13.1	13.4	0.4	0.25
Pigs weaned/litter	10.7	10.3	10.7	0.3	0.26

^ab^Means within a row with different superscripts differ (*P* < 0.05).

^1^Diets containing 125 ppm supplemental Zn as AvailaZinc and ZnSO_4_·H_2_O.

^2^Diets containing 365 ppm supplemental Zn as Control + ZnSO_4_·H_2_O.

^3^Diets containing 595 ppm supplemental Zn as Control + ZnSO_4_·H_2_O.

^4^Calculated as: (number of sows mated within 7 d of weaning/total sows at weaning) × 100; Chi-square = 1.46, df = 2.

^5^Body condition scores evaluated at last rib via caliper.

^6^Initiation of dietary treatments.

^7^Initial body condition score was included as a covariate in the statistical model for subsequent body condition scores.

^8^One day before expected farrowing date.

**Table 4.** Effect of supplemental Zn in late gestation on total number of stillborn and mummified piglets

	Treatment				
Item	CON^1^	INT^2^	HI^3^	Chi-square	*P*-value
Stillborns	76	68	78	8.56^4^	0.20
Mummies	50	49	44	6.37^5^	0.61

^1^Diets containing 125 ppm supplemental Zn as AvailaZinc and ZnSO_4_·H_2_O.

^2^Diets containing 365 ppm supplemental Zn as Control + ZnSO_4_·H_2_O.

^3^Diets containing 595 ppm supplemental Zn as Control + ZnSO_4_·H_2_O.

^4^df = 6.

^5^df = 8.

Sows consuming the INT diet were less likely (*P* < 0.01) to produce pigs with low birth weights (≤1 kg) compared with sows consuming CON or HI treatments (Table 5). Sows producing litters of pigs with a high proportion of low birth weight piglets are typically at greater risk of preweaning mortality (Milligan et al., 2002; Kapell et al., 2011). The number of low birth weight pigs for sows consuming CON or HI treatments in this experiment was greater than those observed in a previous experiment conducted by Bergstrom et al. (2009), where incidence of small pigs per litter weighing less than 1 kg ranged from 8% to 13%. However, the number of low birth weight pigs for sows fed CON and HI treatments was similar to the 14.9% reported by Feldpausch et al. (2019). Overall, it appears that sows used in this experiment produced pigs similar to herds studied previously and were representative of commercial swine production in the United States.

**Table 5.** Effect of supplemental Zn in late gestation on total number of low birth weight piglets

	Treatment				
Item	CON^1^	INT^2^	HI^3^	Chi-square^4^	*P*-value
Low birth weight (≤1.00 kg)	240	165	231	10.78	<0.01
Normal birth weight (≥1.01 kg)	1,325	1,259	1,294		
Total pigs born	1,565	1,424	1,525		
Incidence of low birth weight, %^5^	15.3	11.6	15.1		

^1^Diets containing 125 ppm supplemental Zn as AvailaZinc and ZnSO_4_·H_2_O.

^2^Diets containing 365 ppm supplemental Zn as Control + ZnSO_4_·H_2_O.

^3^Diets containing 595 ppm supplemental Zn as Control + ZnSO_4_·H_2_O.

^4^df = 2.

^5^Calculated as (number of low birth weight pigs/total pigs born) × 100.

### Piglet Growth Performance

Overall, piglets from sows fed the INT treatment had heavier birth weights than piglets from sows receiving CON treatment (Table 6). Although piglets from sows fed INT were heavier at birth, there were no differences in weaning weight or total piglet gain across treatments. As a result, the advantage in initial birth weight for INT piglets was not maintained throughout the nursing period until weaning.

**Table 6.** Effect of supplemental Zn in late gestation on piglet performance

	Treatment				
Item	CON^1^	INT^2^	HI^3^	SE	*P*-value
Overall					
Piglet birth weight, kg	1.38^a,x^	1.42^b^	1.40^ab,y^	<0.01	<0.01
Piglet gain, g/d	227.0	226.5	229.7	1.5	0.28
Piglet weaning weight, kg	5.52	5.59	5.51	0.03	0.14
Piglet age at weaning, d	18.2	18.1	18.1	<0.1	0.44
Total piglet gain, g	4,100	4,140	4,080	30	0.23
Low birth weight (≤1.00 kg)					
Piglet birth weight, kg	0.83	0.84	0.83	<0.01	0.75
Piglet gain, g/d	187.3	190.1	187.9	3.6	0.86
Piglet weaning weight, kg	4.41	4.44	4.34	0.06	0.58
Piglet age at weaning, d	18.9	18.8	18.5	0.1	0.12
Total piglet gain, g	3,532	3,559	3,475	67	0.66
Normal birth weight (1.01 to 1.75 kg)					
Piglet birth weight, kg	1.38^a^	1.41^b^	1.38^a^	<0.01	<0.01
Piglet gain, g/d	227.0	230.1	225.6	1.7	0.16
Piglet weaning weight, kg	5.50^ab^	5.58^a^	5.46^b^	0.03	0.02
Piglet age at weaning, d	18.2	18.2	18.1	<0.1	0.42
Total piglet gain, g	4,112^xy^	4,168^x^	4,073^y^	31	0.09
Heavy birth weight (≥1.76 kg)					
Piglet birth weight, kg	1.94	1.94	1.96	0.01	0.26
Piglet gain, g/d	255.9	250.2	254.9	3.9	0.58
Piglet weaning weight, kg	6.42	6.29	6.44	0.07	0.28
Piglet age at weaning, d	17.6	17.5	17.6	0.1	0.75
Total piglet gain, g	4,485	4,349	4,473	69	0.33

^ab^Means within a row with different superscripts differ (*P* < 0.05).

^xy^Means within a row with different superscripts differ (*P* < 0.10).

^1^Diets containing 125 ppm supplemental Zn as AvailaZinc and ZnSO_4_·H_2_O.

^2^Diets containing 365 ppm supplemental Zn as Control + ZnSO_4_·H_2_O.

^3^Diets containing 595 ppm supplemental Zn as Control + ZnSO_4_·H_2_O.

Growth performance of piglets was evaluated in 3 birth weight categories: Low, Normal, and Heavy. Of all piglets initially identified at birth, 106 pigs did not retain ear tags throughout the entire lactation period, so they were not included in the final data analysis. Therefore, preweaning growth performance and survivability data represent 97.7% of all pigs initially identified (4,514 pigs in total). Total weight gain and weaning weight of low birth weight pigs were similar across dietary treatments. Furthermore, growth rate of heavy birth weight pigs was not different, regardless of the sow’s dietary treatment. However, considering the normal birth weight category, piglets from sows fed INT in late gestation had heavier birth weights compared with piglets from CON or HI sows. The advantage of heavier birth weights for INT pigs (*P* < 0.05) was maintained at weaning compared with pigs farrowed by HI sows, with pigs produced by CON sows being intermediate to the 2 treatments. As a result, preweaning growth performance of piglets tended to improve when sows consumed diets containing 365 ppm of supplemental Zn in late gestation, in contrast to growth rate of pigs from sows consuming diets with 595 ppm of supplemental Zn.

### Preweaning Piglet Mortality

Preweaning mortality of piglets tended to decrease when sows were fed increasing levels of supplemental Zn in late gestation (*P* < 0.10; Table 7). Mortality of low birth weight pigs decreased by 10 percentage points (38.3% to 28.1%) when supplemental Zn was included at 595 ppm compared with 125 ppm (*P* = 0.05). Vallet et al. (2014) also reported reduced preweaning mortality of low birth weight piglets when they fed high dietary Zn to gilts in late gestation. Our results appear to confirm that high supplemental Zn in late gestation may play a role in enhanced survivability of small piglets. Not only did mortality of low birth weight piglets decline, but mortality of heavy birth weight pigs also decreased (*P* < 0.10) due to increased Zn supplementation of sows. Although we hypothesized that survival of low birth weight pigs would improve, we did not expect to observe improvements in survival of heavy piglets.

**Table 7.** Effect of supplemental Zn in late gestation on preweaning mortality of pigs by treatment and weight classification^1^

	Treatment				
Item	CON^2^	INT^3^	HI^4^	Chi-square^5^	*P*-value
Piglet mortality					
Overall				5.41	0.07
Dead^6^	235	188	186		
Alive^7^	1,330	1,236	1,339		
Total pigs	1,565	1,424	1,525		
Mortality, %	15.0	13.2	12.2		
Low birth weight (≤1.00 kg)				5.94	0.05
Dead^6^	92	60	65		
Alive^7^	148	105	166		
Total pigs	240	165	231		
Mortality, %	38.3	36.4	28.1		
Normal birth weight (1.01 to 1.75 kg)				0.11	0.94
Dead^6^	127	120	112		
Alive^7^	987	938	909		
Total pigs	1,114	1,058	1,021		
Mortality, %	11.4	11.3	11.0		
Heavy birth weight (≥1.76 kg)				5.20	0.07
Dead^6^	16	8	9		
Alive^7^	195	193	264		
Total pigs	211	201	273		
Mortality, %	7.6	4.0	3.3		

^1^Data presented as counts of pigs.

^2^Diets containing 125 ppm supplemental Zn as AvailaZinc and ZnSO_4_·H_2_O.

^3^Diets containing 365 ppm supplemental Zn as Control + ZnSO_4_·H_2_O.

^4^Diets containing 595 ppm supplemental Zn as Control + ZnSO_4_·H_2_O.

^5^df = 2.

^6^Represents dead pigs from birth to weaning and does not include stillborn pigs.

^7^Represents live piglets from birth to weaning.

The overall preweaning mortality of pigs in this trial was 13.5%, which is similar to that observed in other commercial facilities (Feldpausch et al., 2019), and 34.1% for low birth weight piglets (Table 8). Mortality of low birth weight piglets was slightly greater than that observed in other commercial facilities, but was not unusual (Bergstrom et al., 2009; Zeng et al., 2019).

**Table 8.** Preweaning mortality of pigs by weight classification^1^

Item	
Overall	
Total deaths^2^	609
Total alive^3^	3,905
Total pigs	4,514
Mortality, %	13.5
Low birth weight (≤1.00 kg)	
Total deaths^2^	217
Total alive^3^	419
Total pigs	636
Mortality, %	34.1
Normal birth weight (1.01 to 1.75 kg)	
Total deaths^2^	359
Total alive^3^	2,834
Total pigs	3,193
Mortality, %	11.2
Heavy birth weight (≥1.76 kg)	
Total deaths^2^	33
Total alive^3^	652
Total pigs	685
Mortality, %	4.8

^1^Data presented as counts of pigs.

^2^Represents dead pigs from birth to weaning and does not include stillborn pigs.

^3^Represents live piglets from birth to weaning.

Zinc is a structural component in many enzymes and metabolic pathways essential for healthy pregnancy and proper function of maternal components such as placental and fetal development. Superoxide dismutase, a Zn-dependent enzyme, provides antioxidant defense for the placenta (Mistry and Williams, 2011), which may have reduced activity during instances of pregnancy complications such as stillbirth in humans. Furthermore, Zn ions are present at the active site of carbonic anhydrase, which is necessary for transport and regulation of carbon dioxide (Tu et al., 2012). Vallet et al. (2014) hypothesized that additional Zn may increase carbonic anhydrase activity which may, in turn, build resistance to high CO_2_ concentrations during birth, and potentially improve chance of survival for piglets that would otherwise be stillborn. Piglets born at the end of the litter are at greater risk for asphyxiation or oxygen deficiency compared with piglets born earlier. Uterine contractions toward the end of parturition reduce the supply of oxygen to the fetus (Alonso-Spilsbury et al., 2005). Vallet et al. (2014) also theorized that additional dietary Zn fed to the dam may potentiate brain myelination of piglets and enhance preweaning piglet survival, but they did not detect any such differences. Researchers and producers are aware that newborn piglets are iron deficient at birth (Matrone et al., 1960; Ullrey et al., 1960), but optimal Zn status of piglets at birth has not been established. Therefore, high supplemental Zn in late gestation may affect Zn status of low birth weight pigs such that chances of survival improve.

High supplemental Zn in late gestation may increase concentrations of metallothionein, an intracellular Zn binding protein, in red blood cells. Metallothionein may be an indicator for assessing Zn status during pregnancy or in periods of stress or trauma (Golden, 1989; Caulfield et al., 2008). Caulfield et al. (2008) suggested that increased erythrocyte concentrations of metallothionein might support rapid proliferation and differentiation of red blood cells and provide protection from oxidative stress that is typically associated with increased oxygen demand during pregnancy. These responses may play a role in mitigating some of the detrimental effects of reduced oxygen supply to the fetus, so that there might be less overall oxygen deprivation. Therefore, the pig may be better prepared for survival outside of the maternal environment.

Most studies have compared clinical presentation of zinc deficiencies during the entire gestation period, but fetal zinc demand may vary throughout gestation. Concentration of fetal liver zinc (about 130 ppm) is greater than sow liver zinc (about 50 ppm). Furthermore, concentration of fetal liver zinc declines until day 70 of gestation and then increases as maternal liver zinc decreases around day 90 of gestation (Hostetler and Kincaid, 2004). Transitory reduced zinc intake during midgestation (0 vs. 100 ppm) decreased litter size and increased postnatal mortality of rat pups (Hurley and Mutch, 1973). Consequently, additional zinc is likely necessary to meet zinc demand of developing fetuses. Investigating effects of high dietary Zn in late gestation on piglet and sow metallothionein concentrations and immunocompetence throughout pregnancy and at birth may guide scientists to answers regarding Zn status of piglets and the observed reduction in mortality of small and heavy birth weight piglets.

### Postweaning Pig Performance and Carcass Characteristics

Postweaning mortality of pigs ranged from 7.6% to 9.6%, similar to wean-to-finish mortality observed in commercial swine facilities (Stalder, 2018). But, postweaning mortality was not different across treatments (Table 9). Late gestation dietary Zn treatments had no effect on carcass characteristics of pigs, regardless of birth weight category (Table 10). There were no significant interactions between dietary Zn treatments in late gestation and birth weight categories for any postweaning performance traits or carcass characteristics. No studies have fully investigated the effects of increased supplemental Zn in late gestation on lifetime performance of offspring. Nonetheless, it appears that additional Zn in late gestation did not affect postweaning lean gain or carcass characteristics. Pigs born at low birth weights often exhibit reduced carcass quality and value at harvest (Rehfeldt et al., 2008; Fix et al., 2010). However, low birth weight pigs from this study performed similar to contemporary pigs of normal birth weights, regardless of dietary treatment. Clearly, increased survival of low birth weight pigs in this experiment did not compromise carcass composition or risk economic losses that one may expect to occur with low birth weight pigs.

**Table 9.** Effects of supplemental Zn in late gestation on postweaning mortality of pigs^1^

	Treatment				
Item	CON^2^	INT^3^	HI^4^	Chi-square^5^	*P*-value
Overall				0.46	0.80
Dead^6^	13	11	11		
Alive^7^	122	134	130		
Total pigs	135	145	141		
Mortality	9.6%	7.6%	7.8%		
Low birth weight (≤1.00 kg)				2.50	0.29
Dead^6^	3	4	1		
Alive^7^	26	23	31		
Total pigs	29	27	32		
Mortality	10.3%	14.8%	3.1%		
Normal birth weight (1.01 to 1.75 kg)				1.48	0.48
Dead^6^	7	6	8		
Alive^7^	61	100	77		
Total pigs	68	106	85		
Mortality	10.3%	5.7%	9.4%		
Heavy birth weight (≥1.76 kg)				<0.01	0.99
Dead^6^	3	1	2		
Alive^7^	35	11	22		
Total pigs	38	12	24		
Mortality	7.9%	8.3%	8.3%		

^1^Data presented as counts of pigs.

^2^Offspring from sows fed a diet containing 125 ppm supplemental Zn as AvailaZn and ZnSO_4_·H_2_O.

^3^Offspring from sows fed a diet containing 365 ppm supplemental Zn as Control + ZnSO_4_·H_2_O.

^4^Offspring from sows fed a diet containing 595 ppm supplemental Zn as Control + ZnSO_4_·H_2_O.

^5^df = 2.

^6^Represents piglets that died from weaning to market.

^7^Represents live piglets from weaning to market.

**Table 10.** Effect of supplemental Zn in late gestation on carcass characteristics of pigs

	Treatment				
Item	CON^1^	INT^2^	HI^3^	SE	*P*-value
Overall					
No. of pigs	122	134	130	—	—
Wean to slaughter, d	168.6	168.3	167.1	1.1	0.59
Hot carcass weight, kg	99.9	101.2	100.1	0.6	0.19
Backfat depth, mm	16.3	16.3	16.0	0.4	0.74
Loin depth, cm	6.9^xy^	6.8^x^	7.0^y^	<0.1	0.07
Lean^4^, %	57.2	57.1	57.5	0.2	0.42
FFL^5^, %	53.5	53.5	53.8	0.2	0.54
Lean gain^6^, g/d	314.5	319.4	320.3	2.6	0.23
Low birth weight (≤1.00 kg)					
No. of pigs	26	23	31	—	—
Wean to slaughter, d	174.0	169.5	171.8	2.1	0.41
Hot carcass weight, kg	97.7	99.8	98.4	1.3	0.57
Backfat depth, mm	15.7	15.9	16.7	0.7	0.55
Loin depth, cm	6.7	6.5	6.8	0.1	0.26
Lean^4^, %	57.3	56.9	56.8	0.5	0.76
FFL^5^, %	53.8	53.5	53.1	0.5	0.62
Lean gain^6^, g/d	301.5	315.3	305.1	6.0	0.25
Normal birth weight (1.01 to 1.75 kg)					
No. of pigs	61	100	77	—	—
Wean to slaughter, d	169.1	168.3	167.2	1.4	0.66
Hot carcass weight, kg	100.1	101.6	101.1	0.8	0.39
Backfat depth, mm	16.7	16.5	15.9	0.5	0.46
Loin depth, cm	7.0	6.9	7.1	0.1	0.35
Lean^4^, %	57.1	57.1	57.7	0.3	0.31
FFL^5^, %	53.4	53.4	53.9	0.3	0.35
Lean gain^6^, g/d	313.7	319.8	323.5	4.8	0.13
Heavy birth weight (≥1.76 kg)					
No. of pigs	35	11	22	—	—
Wean to slaughter, d	163.7	165.8	160.2	2.2	0.28
Hot carcass weight, kg	101.0	101.3	98.8	1.3	0.33
Backfat depth, mm	16.3	14.9	15.2	0.9	0.53
Loin depth, cm	7.0	6.7	7.2	0.1	0.19
Lean^4^, %	57.3	57.8	58.2	0.6	0.55
FFL^5^, %	53.5	54.2	54.3	0.4	0.47
Lean gain^6^, g/d	325.3	324.2	330.1	4.3	0.74

^xy^Means within a row with different superscripts differ (*P* < 0.10).

^1^Offspring from sows fed a diet containing 125 ppm supplemental Zn as AvailaZn and ZnSO_4_·H_2_O.

^2^Offspring from sows fed a diet containing 365 ppm supplemental Zn as Control + ZnSO_4_·H_2_O.

^3^Offspring from sows fed a diet containing 595 ppm supplemental Zn as Control + ZnSO_4_·H_2_O.

^4^Lean calculated as: 58.86 − 0.61 × (backfat depth, in.) + 0.12 × (loin depth, in.) (JBS Pork).

^5^Fat-free lean (FFL) calculated as: [15.31 − (31.277 × backfat depth, in.) + (3.813 × loin depth, in.) + (0.51 × hot carcass weight, lb)]/hot carcass weight × 100; NPPC (2000).

^6^Lean gain calculated as: (FFL at ending weight − FFL in feeder pig)/days on test; NPPC (2000).

Feeding very high concentrations of zinc to sows in late gestation could potentially increase excretion of Zn in feces, which has been previously established for nursery and finishing pigs (Creech et al., 2004). High Zn concentrations in slurry that is later applied to cropland can be detrimental to soil and water quality (Carpenter et al., 1998; Jongbloed and Lenis, 1998; Long et al., 2003). Therefore, utilizing supplemental Zn sources in gestating sow diets that minimize Zn excretion and optimize Zn utilization should be considered.

## CONCLUSION

Results of this experiment indicate that increasing supplemental dietary Zn intake of sows in the last 30 d of gestation decreased overall piglet mortality, mortality of low birth weight piglets, and mortality of heavy birth weight piglets. Subsequent growth performance and carcass characteristics of low birth weight pigs were similar to pigs from sows that did not receive increased supplemental Zn in late gestation. Therefore, there may be substantial value in utilizing increased supplemental Zn in late-gestation sow diets to maximize piglets’ chance of survival. However, further research evaluating sources of supplemental Zn that minimize fecal excretion and total barn output of fecal Zn must be considered.
